# Inhibition of miR‐93‐5p promotes osteogenic differentiation in a rabbit model of trauma‐induced osteonecrosis of the femoral head

**DOI:** 10.1002/2211-5463.13218

**Published:** 2021-07-16

**Authors:** Ying Zhang, Zhikun Zhuang, Qiushi Wei, Peifeng Li, Jitian Li, Yanan Fan, Leilei Zhang, Zhinan Hong, Wei He, Haibin Wang, Youwen Liu, Wuyin Li

**Affiliations:** ^1^ Medical Center of Hip Luoyang Orthopedic‐Traumatological Hospital (Orthopedics Hospital of Henan Province) China; ^2^ Guangzhou University of Chinese Medicine China; ^3^ Institute of Orthopaedics of Guangzhou University of Chinese Medicine China; ^4^ The Third Affiliated Hospital of Guangzhou University of Chinese Medicine China

**Keywords:** bone marrow stromal cells, miR‐93‐5p, osteogenesis, TIONFH

## Abstract

Trauma‐induced osteonecrosis of the femoral head (TIONFH) is characterized by femoral head collapse accompanied by degenerative changes of the hip. We previously reported that miR‐93‐5p expression is abnormally high in patients with TIONFH, but the role of miR‐93‐5p in the TIONFH process remains unclear. Herein, we investigated the role of miR‐93‐5p in TIONFH in a rabbit model. Bone marrow mesenchymal stem cells (BMSCs) were used for both *in vivo* and *in vitro* experiments. A rabbit model of TIONFH was injected with BMSCs transfected with miR‐93‐5p inhibitor. In addition, both an miR‐93‐5p mimic and negative control were transfected into BMSCs. Expression of miR‐93‐5p was significantly increased in the model group compared with control samples. An miR‐93‐5p inhibitor induced the expression of bone morphogenetic protein 2 (BMP‐2) and alkaline phosphatase. Furthermore, expression of osteogenesis‐related markers (BMP‐2, secreted phosphoprotein 1, RUNX family transcription factor 2 and Osterix) was higher in the miR‐93‐5p inhibitor group, as revealed by quantitative PCR and western blotting. In addition, *in vitro* experimentation revealed that an miR‐93‐5p mimic decreased BMP‐2 and TNF receptor superfamily member 11b expression, but increased receptor activator of nuclear factor‐kappaB ligand expression. In summary, the miR‐93‐5p inhibitor could promote osteogenic differentiation by increasing BMP‐2 expression during the development of TIONFH. Thus, miR‐93‐5p may have potential as a therapeutic target for TIONF treatment.

## Graphical abstract

Here, we investigated the role of miR‐93‐5p in a rabbit trauma‐induced osteonecrosis of the femoral head model and *in vitro* using bone marrow mesenchymal stem cells. *In vivo*, miR‐93‐5p expression was significantly increased in the model group, and its inhibition resulted in an increase in the level of osteogenesis‐related markers. *In vitro*, application of a miR‐93‐5p mimic led to a decrease in bone morphogenetic protein 2 expression. Thus, inhibition of miR‐93‐5p may promote osteogenic differentiation in trauma‐induced osteonecrosis of the femoral head.

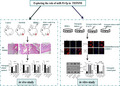

**Abbreviations**
ALP: alkaline phosphatase
BMP‐2: bone morphogenetic protein 2
BMSC: bone marrow mesenchymal stem cell
IHC: immunohistochemistry
miRNA: microRNA
MRI: magnetic resonance imaging
NC: negative control
ONFH: osteonecrosis of the femoral head
OPG: TNF receptor superfamily member 11b
OPN: secreted phosphoprotein 1
qPCR: quantitative PCR
RANKL: receptor activator of nuclear factor-kappaB ligand
RUNX-2: RUNX family transcription factor 2
SD: standard deviation
TBS: Tris-buffered saline
TIONFH: trauma-induced osteonecrosis of the femoral head

Osteonecrosis of the femoral head (ONFH) is a frequently occurring disease, characterized by femoral head collapse accompanied by degenerative changes of the hip [1]. Trauma‐induced ONFH (TIONFH) is a common type of ONFH, which is preceded by traumatic hip dislocation, femoral neck fracture or slipped capital femoral epiphysis [2]. Without timely and effective treatment, patients with TIONFH may be left with permanent disability. Reportedly, patients with TIONFH can be treated with core decompression of the hip and total replacement of the hip [3]; however, the long‐term prognosis is poor [4]. Therefore, there is urgent need to find biological approaches to treat TIONFH.

Growing evidences have revealed that the ability of bone marrow mesenchymal stem cells (BMSCs) to differentiate plays a crucial role in the treatment of patients with ONFH [5, 6, 7]. Indeed, BMSCs have shown significant osteogenic potential in various animal models of bone repair [8, 9], and stem cell therapies have been applied to clinical treatment of ONFH [10]. A recent meta‐analysis has reported that BMSC implantation has a positive therapeutic effect on patients with ONFH [11]. In the past few years, microRNAs (miRNAs), a type of small noncoding RNA with the ability to repress gene expression, are increasingly recognized as playing a functional role in the pathogenesis and treatment of ONFH. Dai *et al*. [12] indicated that miR‐217 promoted osteogenic differentiation via inhibition of the Dickkopf WNT signaling pathway inhibitor 1 during the development of steroid‐induced ONFH. Moreover, a study reported by Liao *et al*. [13] observed that overexpression of miR‐122‐5p might down‐regulate sprouty RTK signaling antagonist 2, leading to alleviate the development of ONFH. However, the role of miRNA in the pathogenesis of TIONFH remains poorly understood.

In our former study, we examined abnormal miRNAs in patients with TIONFH and identified a total of 35 up‐regulated miRNAs (including miR‐93‐5p) [14]. Subsequently, cell culture experiments were conducted and confirmed that up‐regulation of miR‐93‐5p inhibited osteogenic differentiation by reducing the expression level of bone morphogenetic protein 2 (BMP‐2) during the development of TIONFH. In previous studies, miR‐93‐5p has been reported in various types of cancers, including gastric cancer [15], breast cancer [16] and esophageal cancer [17]. Among these studies, we observe that miR‐93‐5p mainly functions in cell migration and invasion. Thus, we hypothesize that inhibiting miR‐93‐5p may promote osteogenic differentiation, but the exact mechanism remains unclear.

In this study, we used a rabbit model *in vivo* and BMSCs *in vitro* to research the role of miR‐93‐5p in the pathogenesis of TIONFH, providing potential therapeutic targets for TIONFH.

## Materials and Methods

### Animals model establishment and treatment groups

**Fig. 1:**
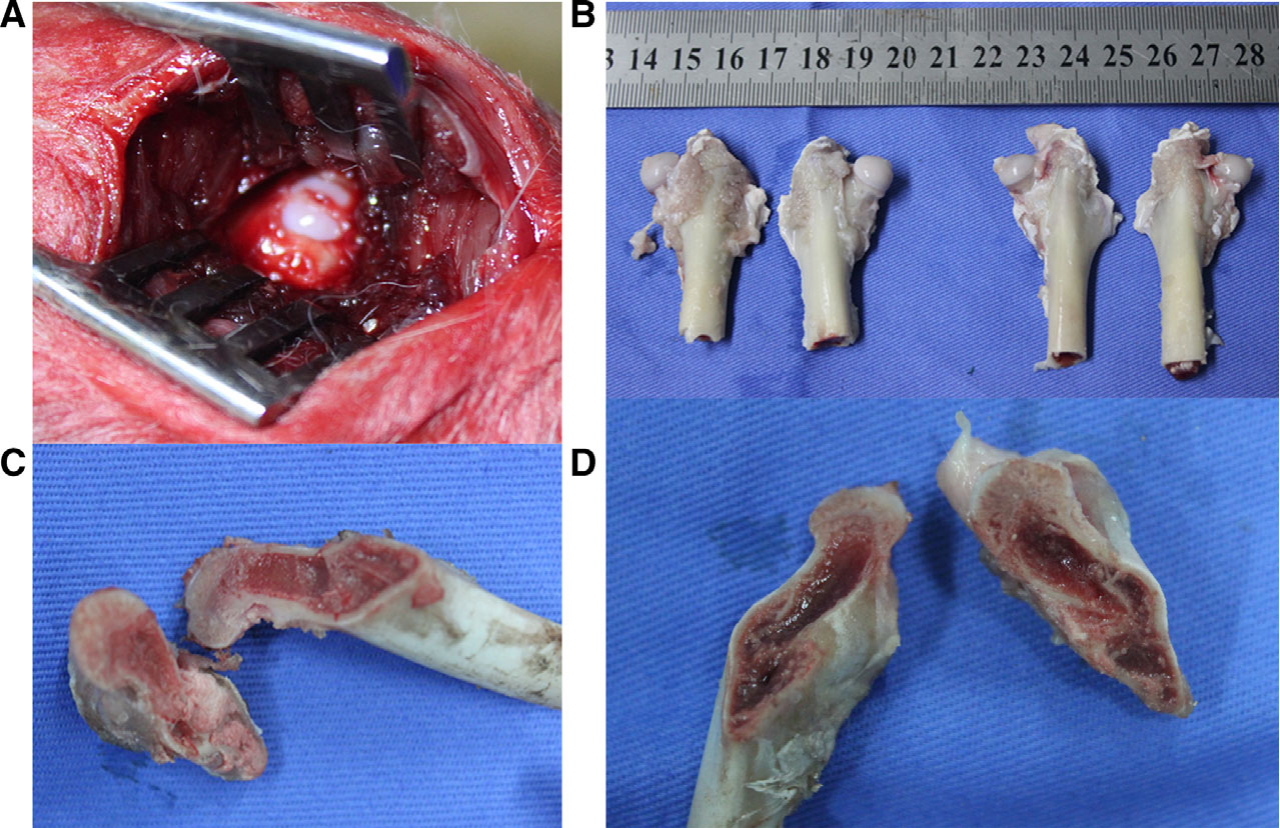
Surgical procedure of TIONFH. (A) Exposed femoral head. (B) Measurement and appearance of the animal femoral head in normal and model groups. (C) Cross‐sectional view of the TIONFH. (D) The cross‐sectional view of the normal femoral head. The region in white is the necrotic area, while the region showing redness is the normal area. We observe that the model side femur is white, presenting ischemic necrosis, and the bone marrow of the normal femur is bright.

The experimental protocols were approved by the Animal Ethics Committee at Guangzhou University of Chinese Medicine, and all processes were performed in accordance with the institutional guidelines for animal care. Thirty‐two healthy New Zealand white rabbits (16 weeks, males and females, 2.8–3.3 kg) were used for experiments after a 7‐day adaptation period under the standard laboratory conditions. Among these, six rabbits were set as controls, and 24 rabbits underwent traumatic surgery. Rabbits were anesthetized with 3% pentobarbital sodium (1 mL·kg^−1^; Sinopharm Chemical Reagent Co., Ltd, Shanghai, China). A posterolateral incision was made in the right hip under aseptic conditions, the joint capsule was cut, and then the femoral head was exposed. Next, a stainless steel bone‐groove knife was used to form an external force fracture in the femoral neck and dislocate the femoral head. Meanwhile, the fracture remained separated for 2–3 min. Subsequently, the femoral head was repositioned and secured with sutures and no external fixation. Finally, the wound was injected with antibiotics before being closed in layers. During the postoperative period, all animals were free to move, and no postoperative infection was observed. The left femoral head was used as control (Fig. 1). The 24 postoperative animals were randomly divided into three equal‐size groups: the model group (rabbits with TIONFH), the model + BMSCs group (TIONFH rabbits injected with BMSCs) and the model + BMSCs/miR‐93‐5p inhibitor group (TIONFH rabbits injected with miR‐93‐5p inhibitor‐transfected BMSCs).

### Magnetic resonance imaging examinations

To identify whether the model was successful, we performed a magnetic resonance imaging (MRI) examination after 2 weeks of treatment. MRI was conducted by a GE Signa EXICITE HD 1.5T superconducting MR machine (GE Medical System, Milwaukee, WI, USA). An axial scan was performed by spin‐echo/T1‐weighted imaging, fast spin‐echo/T1 and a fast‐spin echo/T2 fat suppression sequence using 8‐channel receiver head coils. T1‐weighted axial fast multiplanar spoiled gradient‐echo images from the hip joint were obtained with the following parameters: slice thickness/spacing, 4/0 mm; acquisition matrix, 256 × 192; echo time/repetition time, 9/4 ms; initial time, 200 ms; flip angle 8 degrees; and field of view, 14 × 14 cm^2^.

### Isolation and cultivation of BMSCs

Primary BMSCs were isolated as described previously [18]. In brief, two New Zealand rabbits were anesthetized by injection with 3% pentobarbital sodium (1 mL·kg^−1^), and 10 mL of bone marrow was extracted from the femur using a heparinized syringe. The bone marrow was diluted with Dulbecco's modified Eagle's medium high‐glucose medium (containing 10% FBS) and then centrifuged at 200 ***g*** for 5 min. After removing fat, the cells were resuspended. Next, the cell suspension was centrifuged with 1.073 g·mL^−1^ Percoll lymphocyte separation medium, and the mononuclear cells located at the junction of the liquid level were collected. After washing twice with the sterile PBS, the cells were cultured in Dulbecco's modified Eagle's medium. The cells were seeded in a 100‐mm culture dish and cultured at 37 °C in a cell incubator with 5% CO_2_. Half of the culture medium was exchanged after 36‐h incubation. Cells at 80% confluency were digested with 0.25% trypsin and then centrifuged and passaged at a 1 : 1 ratio. The second generation cells were given an extended cultivation at 1 : 3 ratio. Finally, the third generation cells were harvested for the following experiments, and then stem cells were identified by flow cytometry.

### Identification of the BMSCs

To authenticate the phenotypic character, we used the cell identification kit (Cyagen Biosciences Inc., Guangzhou, China) to collect the third generation BMSCs. Afterward, the expression of surface markers (CD34, CD105, CD90, CD73 and CD45) was detected by flow cytometry. In brief, cells were incubated with FITC‐tag for 10 min and then washed twice with PBST. IgG1 was used as control. The expression of cell antibodies was detected by BD Accuri C6 flow cytometry within 6 h. The experiment was repeated three times.

### Transfection of miR‐93‐5p inhibitor into rabbit BMSCs

The miR‐93‐5p inhibitor (sequence: 5′‐CTACCTGCACGAACAGCACTTTC‐3′) was compounded by RiboBio Co. Ltd (Guangzhou, China) and then transfected into BMSCs using Lipofectamine 2000 according to the manufacturer's instructions.

### Local injection of BMSCs

Two weeks after surgery, the femoral head was re‐exposed to inject BMSCs. For the model + BMSCs group, 5 × 10^6^ BMSCs were injected into model animals. In addition, for the model + BMSCs/miR‐93‐5p inhibitor group, 5 × 10^6^ miR‐93‐5p inhibitor‐ transfected BMSCs were injected into TIONFH animals through the femoral marrow.

### Quantitative PCR analysis of miR‐93‐5p

Peripheral blood of animals in each group was collected for miR‐93‐5p detection at 2 and 4 weeks after treatment. Total RNA samples were extracted using the TRIzol method. A looped antisense primer (5′‐CTCAACTGGTGTCGTGGAGTCGGCAATTCAGTTGAGCTACCTGC‐3′) was used for reverse transcription. Quantitative PCR (qPCR) was carried out according to the manufacturer’s instructions using the mirVana™ qRT‐PCR miRNA Detection Kit (RiboBio Biotechnology, Guangzhou, China), and U6 was selected as an internal control.

### Histological examinations and immunohistochemical detection

After 8 weeks, the animals were sacrificed. The femoral head was sampled and fixed in 4% formaldehyde solution for 48 h. Subsequently, the specimens were decalcified by 10% ethylenediaminetetraacetic acid for 10–15 days. Buffer was changed every 3 days. Then, samples were embedded in paraffin and cut into 5‐μm‐thick sections in the coronal plane.

Some sections were analyzed for morphology using histology. The specimens were deparaffinized by xylol and then rehydrated in successively decreasing grades of ethanol. Subsequently, the sections were stained with hematoxylin (5–20 min) and eosin (30 s) at room temperature. Histological changes were observed under a Leica stereomicroscope (MZ 6) at 100× magnification.

On the remaining sections, immunohistochemistry (IHC) was performed to detect BMP‐2 and alkaline phosphatase (ALP) protein expression. In detail, the sections were dewaxed in xylene, rehydrated in ethanol and washed in Tris‐buffered saline (TBS) twice for 3 min. Afterward, sections were incubated with 3% hydrogen peroxide for 10–15 min and washed with TBS twice for 5 min. Then sections were incubated with sealing solution at 25 °C for 5 min and washed in TBS. The sections of samples were incubated at 37 °C for 2 h with the following primary antibodies: BMP‐2 (dilution 1 : 300, sc‐137087; Santa Cruz Biotechnology, Santa Cruz, CA, USA) and ALP (dilution 1 : 200, sc‐365765; Santa Cruz Biotechnology). After that, sections were washed with TBS twice for 5 min, incubated with Enhancer reagent at room temperature for 20 min and rinsed again with TBS. Finally, the sections were treated with HRP Polymer for 30 min at room temperature and stained with diaminobenzidine. The sections were observed under a Leica stereomicroscope at 100× magnification.

### qPCR assay of osteogenesis‐related genes

The expression level of osteogenesis‐related genes, including BMP‐2, secreted phosphoprotein 1 (OPN), RUNX family transcription factor 2 (RUNX‐2) and Osterix, was detected by qPCR. RNA was extracted from the rabbit's femoral head using TRIzol reagent (Invitrogen Corp., Carlsbad, CA, USA). The synthesis of first‐strand cDNA was performed by PrimeScript™ RT reagent Kit (RR037A; TaKaRa, Dalian, China). The SYBR Premix Ex Taq kit (RR420A; TaKaRa) was used to perform qPCR, according to the manufacturer's protocol, on the ABI7500 FAST real‐time PCR system under the program: 95 °C for 30 s, 40 cycles of 95 °C for 3 s and 60 °C for 30 s. The relative expression of genes was calculated with the 2^−ΔΔ^Ct method, and β‐actin was used for internal control. The primer sequences are listed in Table 1.

**Table 1:** Primer sequences for qPCR.

Gene	Forward (5′–3′)	Reverse (5′–3′)
*BMP2*	GGTGGAATGACTGGATTGT	GAGATAGCACTGAGTTCTGT
*RUNX2*	CAGCACTCCATATCTCTACTAT	CTTCCATCAGCGTCAACA
*Osterix*	CAGGCTATGCTAATGATTACC	GGCAGACAGTCAGAAGAG
*OPN*	ATGGCTTTCATTGGAGTTGCTTG	TGGTTTGCCTTTGCCTGTTCG
*β‐Actin*	ATGCTGCTTACATGTCTCGAT	AGCAGAGAATGGAAAGTCAAA

### Western blotting assay

The protein abundance of osteogenesis‐related markers was also tested by western blotting. The femoral head was crushed in liquid nitrogen using a mortar and dissolved in immunoprecipitation assay buffer, and protein was extracted. The protein concentration was measured using the bicinchoninic acid quantitative assay. Protein samples (20 μg) were separated using 12% SDS/PAGE and transferred onto a polyvinylidene fluoride membrane. Blocking was performed using 5% skim milk powder for 1 h at room temperature. The membrane was incubated with primary antibodies against glyceraldehyde‐3 phosphate dehydrogenase (dilution 1 : 1400, KM9002; Tianjin Sungene Biotech Co., Ltd., Tianjin, China), BMP‐2 (dilution 1 : 1000, sc‐137087; Santa Cruz Biotechnology), RUNX‐2 (dilution 1 : 1000, sc‐101145; Santa Cruz Biotechnology), OPN (dilution 1 : 1000, sc‐21742; Santa Cruz Biotechnology) and Osterix (dilution 1 : 1000, sc‐22538; Santa Cruz Biotechnology) overnight at 4 °C and then incubated with horseradish peroxidase‐labeled secondary antibodies (dilution 1 : 5000) for 50 min at room temperature. After washing with 0.05% TBST, the protein accumulation was detected by the Millipore ECL Western Blotting Detection System (EMD Millipore, Billerica, MA, USA).

### Immunofluorescence detection *in vitro*

miR‐93‐5p mimics were transfected into BMSCs, and nonspecific miRNA was used as negative control (NC). After 48‐h infection, osteogenic inducer (containing 50 mg·L^−1^ ascorbic acid, 0.1 μmol·L^−1^ dexamethasone and 10 mmol·L^−1^ β‐glycerophosphate) was supplemented, and then cells were cultured for 7 days. Next, after removing the culture solution, cells were fixed with 4% paraformaldehyde and permeabilized with 1% Triton X‐100 for 10 min. Transfected cells were washed with PBS and blocked with 3% BSA for 30 min. Subsequently, cells were incubated overnight with BMP‐2 primary antibody (dilution 1 : 1000, ab6285; Abcam, Cambridge, UK) at 4 °C and then incubated with secondary antibody (dilution 1 : 2000, ab150115; Abcam) at room temperature for 30 min. Finally, cells were incubated for 10 min with 4, 6‐diamidino‐2‐phenylindole (10 μg·mL^−1^) for nuclear staining at room temperature. Immunofluorescence was examined using a ZEISS LSM 800 (Carl Zeiss Inc., Thornwood, NY, USA) at 200× magnification.

Furthermore, in order to detect the expression levels of several key osteogenic‐related proteins in cells, total protein of these cells was isolated using radioimmunoprecipitation assay buffer, and then the protein expression level of TNF receptor superfamily member 11b (OPG) and receptor activator of nuclear factor‐kappaB ligand (RANKL) was determined by using western blotting. The cells were incubated with the following primary antibody at 4 °C for 12 h: anti‐OPG rabbit polyclonal Ig (dilution 1 : 300; Abcam), anti‐RANKL Ig (dilution 1 : 1000, sc‐377079; Santa Cruz Biotechnology) and anti‐glyceraldehyde‐3 phosphate dehydrogenase (dilution 1 : 1400, KM9002; Tianjin Sungene Biotech Co., Ltd.). Next, cells were incubated with secondary antibodies (dilution 1 : 5000) for 50 min at room temperature. The results were acquired using electrophoresis gel imaging (Bio‐Rad, Hercules, CA, USA).

### Statistical analysis

All experiments were repeated three times. Data were presented as means ± standard deviation (SD). The graphpad prism 8.0 (GraphPad Software, Inc., La Jolla, CA, USA) software was used to analyze results. One‐way ANOVA followed by a Dunnett’s multiple comparison test was selected to calculate statistical differences. *P* < 0.05 was considered significant.

## Results

### The appearance of the femoral head

After modeling, we observed the appearance and cross section of the femur. Figure 1A showed the exposed femoral head, and the diameter of the femoral head was larger in the control group than in the model group (Fig. 1B). In addition, the cross‐sectional view showed that the model side femur was white, presenting ischemic necrosis (Fig. 1C), while the bone marrow of the normal femur was bright (Fig. 1D).

### MRI examination of femoral head

**Fig. 2:**
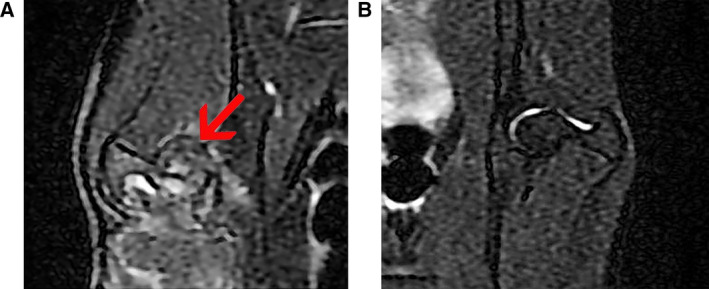
MRI examination of femoral head. (A) MRI of TIONFH. (B) MRI of normal femoral head. The crescents and collapses are indicated by the red arrow.

The image of the right hip showed a double‐line sign accompanied by collapse of the femoral head (Fig. 2A). In addition, a crescent sign also was observed. These characteristics indicated that the TIONFH model was obtained. Figure 2B displays the MRI examination of normal femoral head.

### Identification of BMSCs

**Fig. 3:**
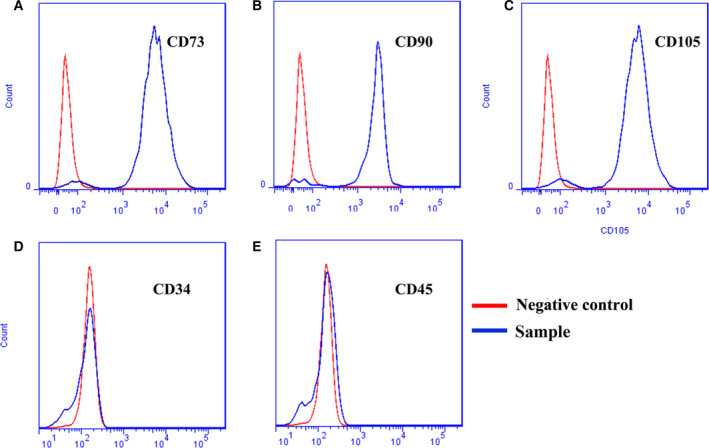
Detection of the surface markers of BMSCs by flow cytometry: (A) CD73, (B) CD90, (C) CD105, (D) CD34 and (E) CD45. Red line represents NC, and blue line represents test sample. Results showed that BMSCs were positive for CD73, CD90 and CD105, but negative for CD45 and CD34.

The results demonstrated that the isolated cell expression was positive for mesenchymal stem cells markers, including CD73, CD90 and CD105 (Fig. 3A–C), and was negative for the hematopoietic marker CD34 (Fig. 3D) and leukocyte marker CD45 (Fig. 3E). The results were in line with BMSC phenotypic characteristics.

### The expression level of miR‐93‐5p

**Fig. 4:**
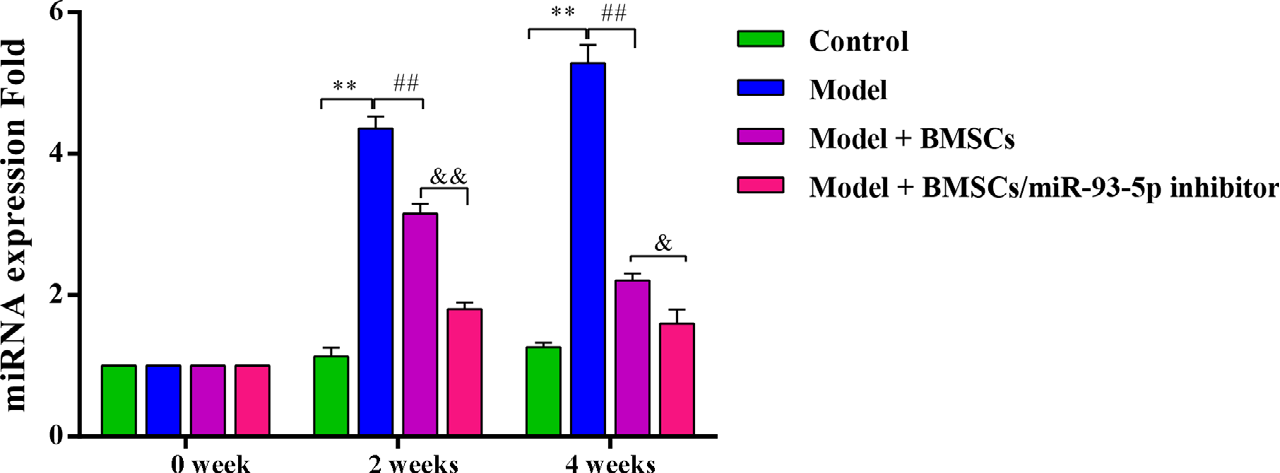
The gene expression of miR‐93‐5p detected by qPCR. There are eight rabbits used for each group (*n* = 8), and data were expressed as mean ± SD. Differences between two groups were determined by one‐way ANOVA. **P < 0.01 vs. the control group; ^##^ *P* < 0.01 vs. the model group; ^&^ *P* < 0.05, ^&&^ *P* < 0.01 vs. the model + BMSCs group.

The mRNA expression level of miR‐93‐5p in the model group was markedly higher than that in the control group (*P* < 0.01), and it was gradually increased in the model group during the treatment process. Ranging from 2 to 4 weeks, a significant decrease of miR‐93‐5p was observed in the model + BMSCs group, and a similar trend was found in the model + BMSCs/miR‐93 5p inhibitor group (all *P* < 0.01). Furthermore, the expression level of miR‐93‐5p was observably lower in the inhibitor group than in the remaining three groups (Fig. 4).

### Histological observations and IHC analysis

**Fig. 5:**
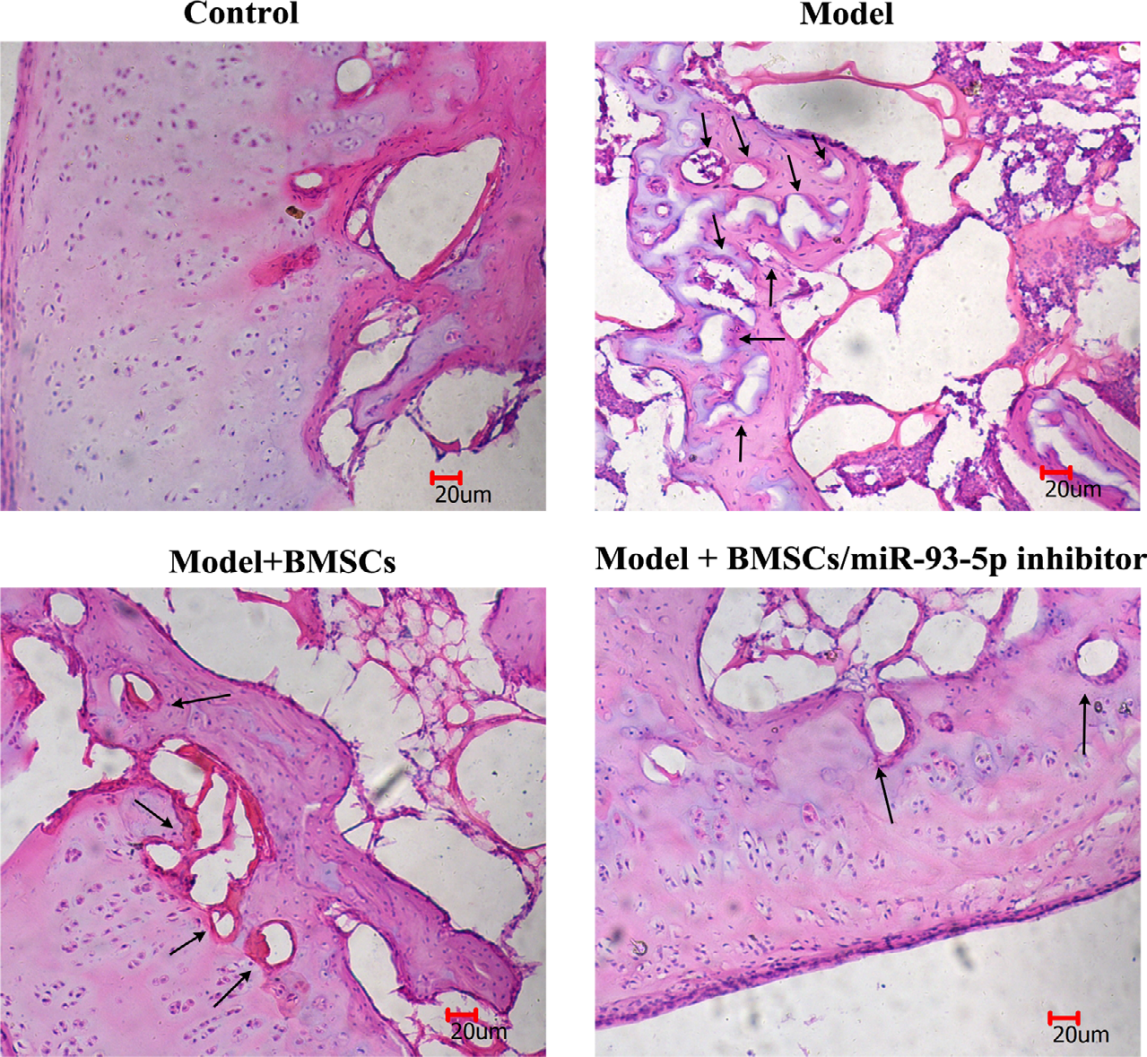
Hematoxylin and eosin staining of rabbit femoral heads in different groups (scale bars: 20 µm; *n* = 8). The model group showed numerous empty lacunae (black arrows) surrounded by necrotic marrow cells. Fewer empty lacunae and more osteoblasts were observed in the model + BMSCs group and model + BMSCs/miR‐93‐5p inhibitor group.

As shown in Fig. 5, the sections stained with hematoxylin and eosin showed the femur was intact without osteoclasts in the normal group, suggesting that the samples were healthy without inflammation. However, obvious osteonecrosis with more empty lacunae was observed in the model group. Compared with the model group, fewer empty lacunae and more osteoblasts were observed in the model + BMSCs group and model + BMSCs/miR‐93‐5p inhibitor group. In addition, we found the number of empty lacunae in the model + BMSCs/miR‐93‐5p inhibitor group was less than in the model + BMSCs groups.

**Fig. 6:**
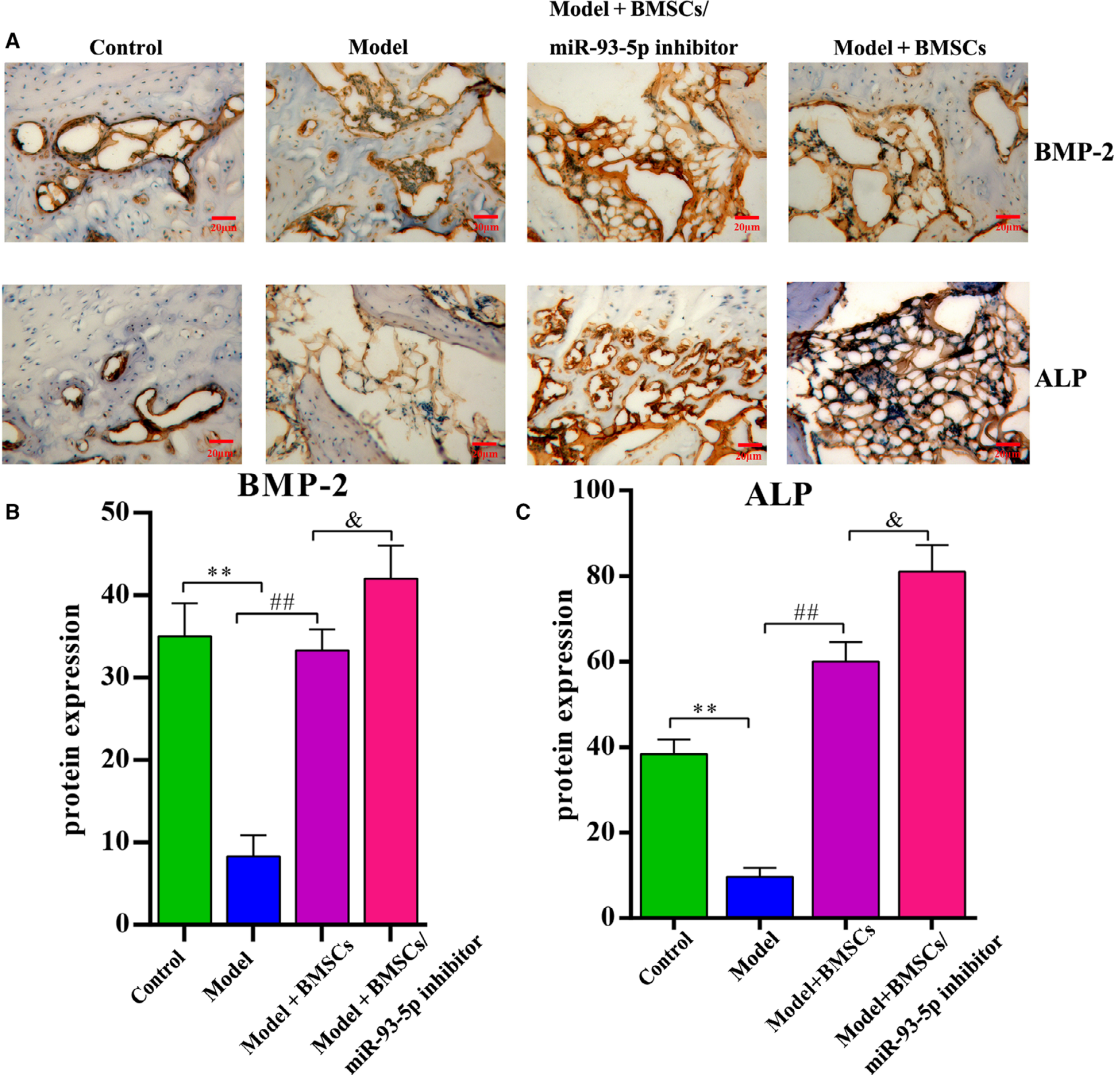
IHC staining of ALP and BMP‐2. (A) ALP and BMP‐2 expression detected by IHC staining (scale bar: 20 µm). Brown represents positive signals. (B) Quantitative analysis of BMP‐2 protein expression level. (C) Quantitative analysis of ALP protein expression level. There were eight animals for each group (*n* = 8), and data were expressed as mean ± SD. Differences between two groups were determined by one‐way ANOVA. **P < 0.01 vs. the control group; ^##^ *P* < 0.01 vs. the model group; ^&^ *P* < 0.05 vs. the model + BMSCs group.

BMP‐2 and ALP protein levels were detected by IHC analysis. The representative image is shown in Fig. 6A. The levels of BMP‐2 (Fig. 6B) and ALP (Fig. 6C) were significantly lower in the model group than in the control group (all *P* < 0.01). The model + BMSCs/miR‐93‐5p inhibitor group exhibited higher levels of BMP‐2 and ALP protein than the model group (*P* < 0.01), suggesting the inhibitor group had higher osteoblast differentiation ability.

### Osteogenesis‐related markers detected by qPCR and western blotting assay

**Fig. 7:**
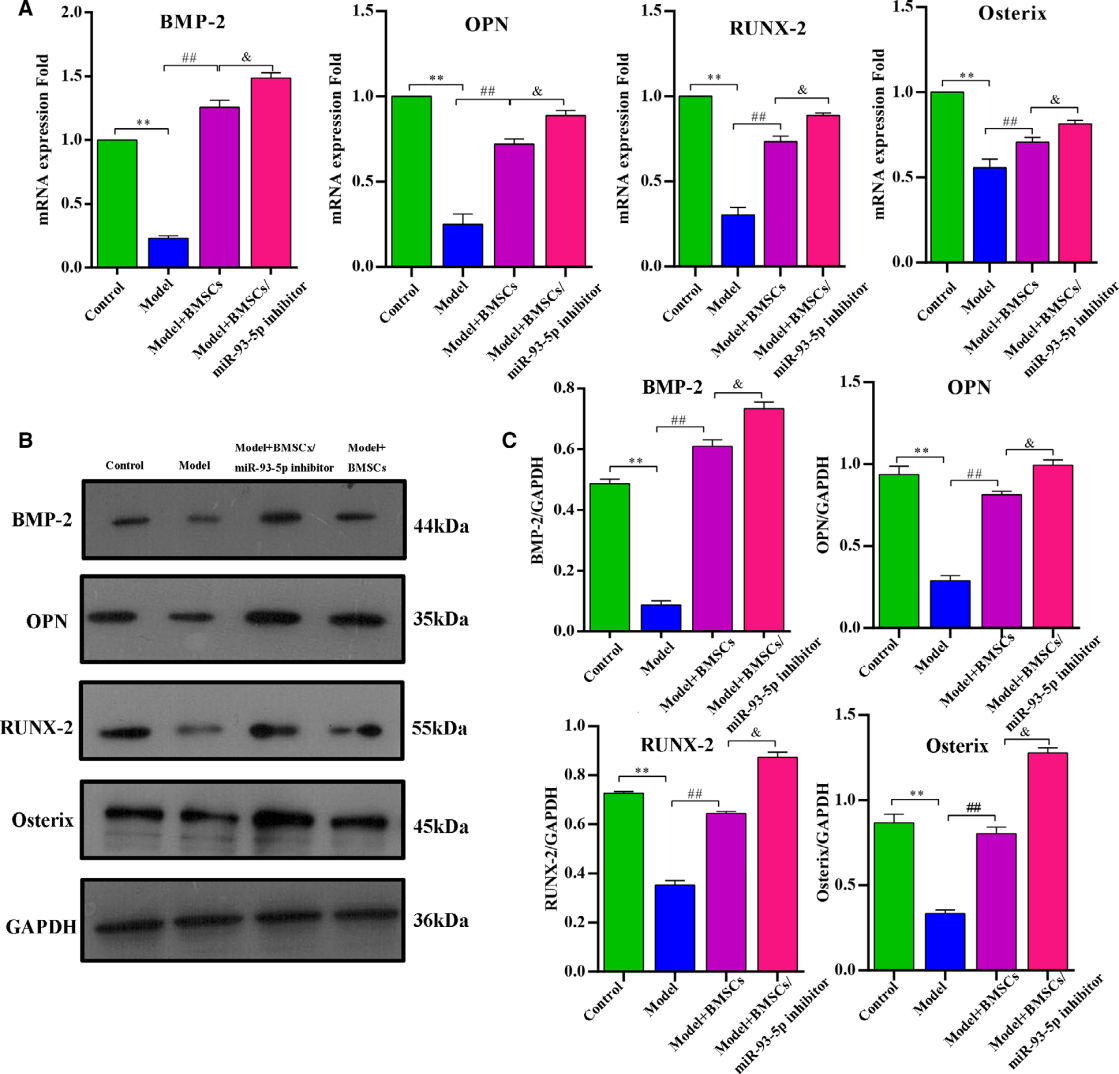
Inhibition of miR‐93‐5p promoted osteogenic differentiation in the TIONFH rabbit. (A) The expression levels of osteogenesis‐related genes (BMP‐2, OPN, RUNX‐2 and Osterix) measured by qPCR. (B) The protein expression level of BMP‐2, OPN, RUNX‐2 and Osterix measured by western blotting. (C) Quantified results of BMP‐2, OPN, RUNX‐2 and Osterix proteins in the different groups. Values are shown as mean ± SD; *n* = 8 in each group. Differences between two groups were determined by one‐way ANOVA. **P < 0.01 vs. the control group; ^##^ *P* < 0.01 vs. the model group; ^&^ *P* < 0.05 vs. the model + BMSCs group.

The mRNA expression level of osteogenesis‐related genes was detected by qPCR. The expression of BMP‐2, which was considered an important factor in stimulating bone formation, was remarkably decreased in the model group compared with the control group (*P* < 0.01). In addition, BMP‐2 was significantly increased in the model + BMSCs/miRNA‐93‐5p inhibitor group compared with the model group (*P* < 0.01). Compared with the model + BMSCs group, BMP‐2 expression level in the model + BMSCs/miR‐93‐5p group was significantly increased (*P* < 0.05). Notably, OPN, RUNX‐2 and Osterix showed similar tendencies as BMP‐2 (Fig. 7A). Furthermore, the expression of osteogenesis proteins was detected by western blotting, and the protein abundance had consistent trend with the qPCR data. In brief, the expression of BMP‐2, OPN, RUNX‐2 and Osterix in the model group was significantly lower than in the control group, which was drastically higher in the model + BMSCs group than in the model group (*P* < 0.01). In addition, we observed that OPN, RUNX‐2 and Osterix were significantly higher in the model + BMSCs group than in the model group (*P* < 0.01), and the level of these proteins was observably increased in the model + BMSCs/miR‐93‐5p inhibitor group than in the model + BMSCs group (*P* < 0.05; Fig. 7B,C).

### Immunofluorescence detection *in vitro*

**Fig. 8:**
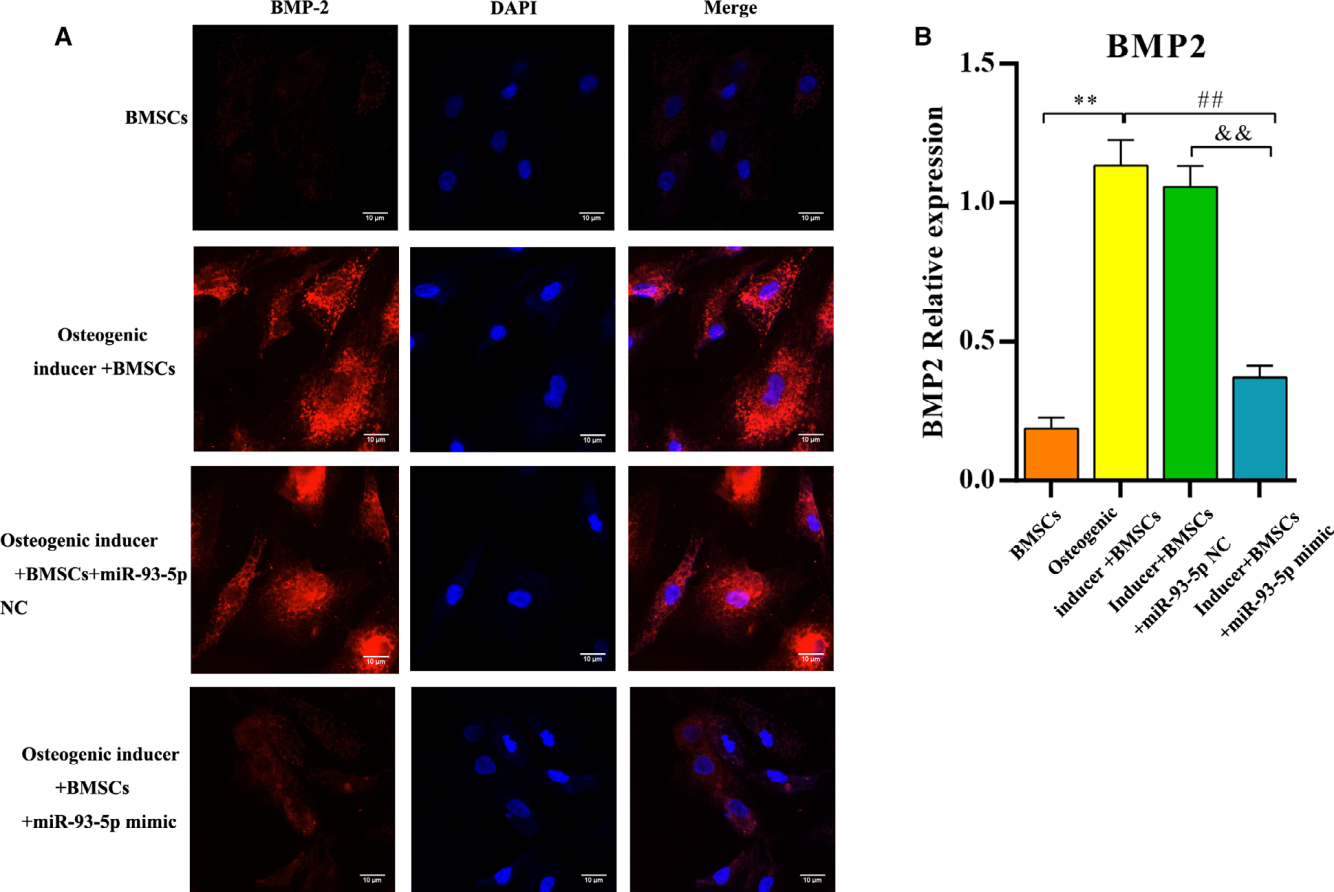
Overexpression of miR‐93‐5p suppressed the BMP‐2 expression level *in vitro*. (A) Immunofluorescence images showing BMP‐2 staining of the cells in the different groups (scale bars: 10 µm). (B) Quantitative analysis of BMP‐2 protein expression level in each group. Values are shown as mean ± SD; *n* = 3 in each group. Differences between two groups were determined by one‐way ANOVA. **P < 0.01 vs. the BMSCs group; ^##^ *P* < 0.01 vs. the osteogenic inducer + BMSCs group; ^&&^ *P* < 0.01 vs. the inducer + BMSCs/miR‐93‐5p NC group.

To determine whether miR‐93‐5p could repress BMP‐2 expression in BMSCs, we transfected the miR‐93‐5p mimic or NC into BMSCs. Immunofluorescence revealed that BMP‐2 expression was induced during osteogenic differentiation of BMSCs (Fig. 8A). In addition, we observed that the BMP‐2 expression was weak in the normal BMSCs group. Compared with the BMSCs group, the protein expression of BMP‐2 was significantly higher in other groups. In addition, the level of BMP‐2 in the inducer + BMSCs/miR‐93‐5p mimic group was significantly lower than the inducer + BMSCs and inducer + BMSCs/miR‐93‐5p NC groups (*P* < 0.01). These findings showed that BMP‐2 was suppressed by miR‐93‐5p mimic transfection, suggesting that impaired differentiation of BMSCs was caused by miR‐93‐5p overexpression (Fig. 8B).

**Fig. 9:**
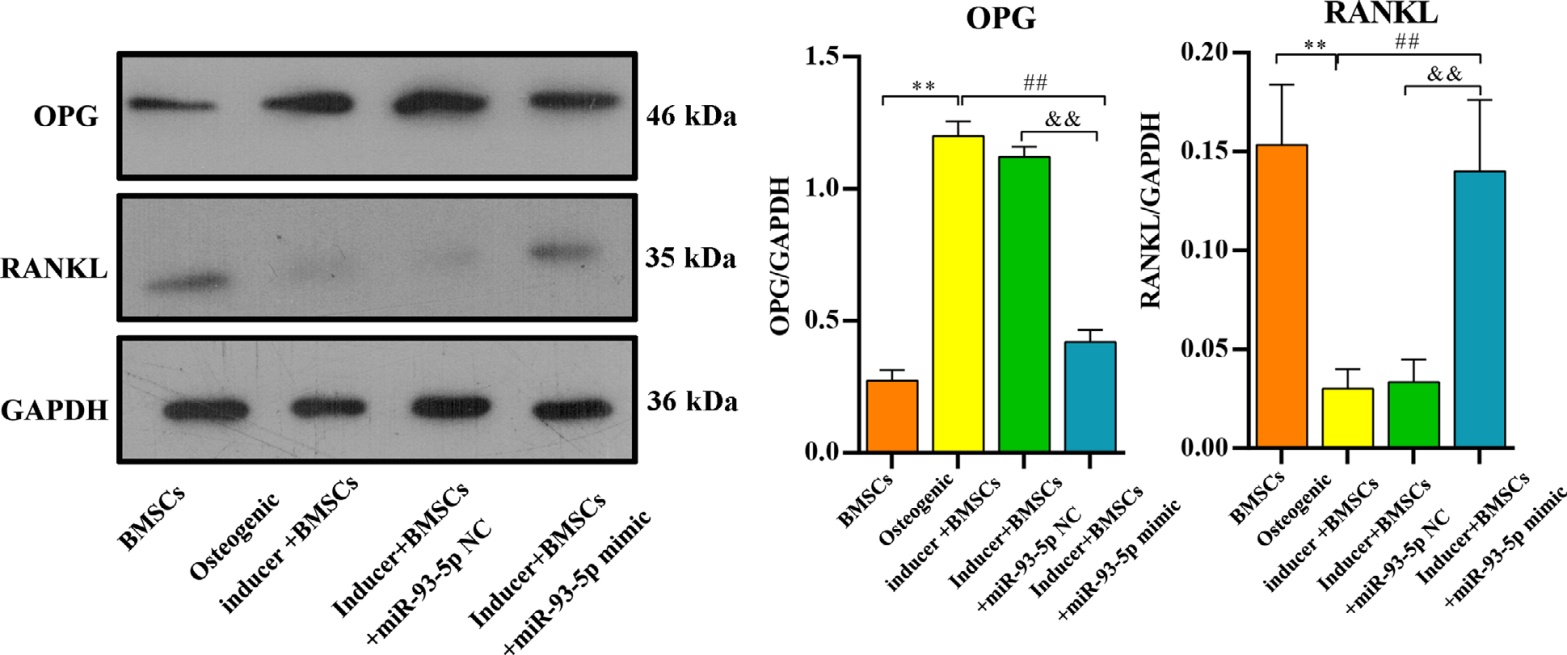
Overexpression of miR‐93‐5p inhibited the OPG expression and increased the RANKL expression. Data are represented as mean ± SD; *n* = 3 in each group. Differences between two groups were determined by one‐way ANOVA. **P < 0.01 vs. the BMSCs group; ^##^ *P* < 0.01 vs. the osteogenic inducer + BMSCs group; ^&&^ *P* < 0.01 vs. the inducer + BMSCs/miR‐93‐5p NC group.

Moreover, we detected the protein expression of OPG and RANKL. Compared with the BMSCs group, the expression of OPG in the inducer + BMSCs and the inducer + BMSCs/miR‐93‐5p NC was significantly increased (*P* < 0.01). However, a decreased OPG was observed in the inducer + BMSCs/miR‐93‐5p mimic group (*P* < 0.01), which almost recovered to normal level. RANKL expression was significantly declined in the inducer + BMSCs and the inducer + BMSCs/miR‐93‐5p NC groups (*P* < 0.01), which was increased by treatment with miR‐93‐5p mimic (Fig. 9).

## Discussion

TIONFH is mainly caused by decreased blood flow to the femoral head and lesion of bone trabecula, resulting in articular cartilage collapse and hip joint dysfunction [19]. Convincing evidence indicates that BMSCs may be critically involved in bone differentiation and tissue regeneration [20, 21]; hence understanding the osteogenic differentiation mechanism of BMSCs is important to provide new insight for increasing treatment options. Additional studies have suggested that osteogenic differentiation and bone remodeling processes are regulated by miRNAs [22, 23, 24].

We previously confirmed that miRNA‐93‐5p suppressed osteogenic differentiation of human BMSCs by targeting BMP‐2 [14], but the specific function of miR‐93‐5p in osteogenic differentiation remained unclear. In this study, the effect of miR‐93‐5p on osteonecrosis was investigated in a rabbit model. We observed that injection of the miR‐93‐5p inhibitor could promote osteogenic differentiation, and the effect of the inhibitor was better than that of the BMSCs group. In addition, with a series of *in vivo* assays, we observed that expression of osteogenesis‐related proteins (BMP‐2, OPN, RUNX‐2 and Osterix) was increased, whereas miR‐93‐5p expression was reduced during the osteogenic differentiation of BMSCs. Furthermore, during *in* *vitro* experiments, addition of miR‐93‐5p mimic in osteoblast‐induced BMSCs resulted in an overexpression of RANKL and low expression of BMP‐2, as well as OPG. All the earlier evidences further supported that BMP‐2 was regulated by miR‐93‐5p during osteogenic differentiation. Moreover, histology and IHC indicated that the effect of bone differentiation in the inhibitor group was better than that in the model and BMSCs groups.

miRNA‐93‐5p, as a member of the miR106b‐25 family, is located on chromosome 11q22.1 [25]. Recently, miRNA‐93‐5p has been widely investigated and reported to play a role in various types of cancer [26, 27] and cell differentiation [28]. Furthermore, emerging findings have demonstrated that the bone remodeling processes and osteogenic differentiation are regulated by miRNA‐93‐5p. Quan *et al*. [29] found that miR‐93‐5p served a crucial role in osteoclastogenesis and vasculogenesis. Meanwhile, Xu *et al*. [30] observed that miR‐93‐5p suppressed osteogenic differentiation of mouse BMSCs by targeting Smad5. In our previous study, an increased miR‐93‐5p was observed in patients with TIONFH, which could inhibit osteogenic differentiation and was associated with BMP‐2 reduction [14]. However, the specific mechanism of miR‐93‐5p that affects BMP‐2 expression is still unclear. In this study, we further confirmed the effect of miR‐93‐5p on osteogenic differentiation using a rabbit TIONFH model. Inhibition of miR‐93‐5p could promote BMSCs osteogenic differentiation and increased the expression of osteogenesis‐related proteins, which was consistent with the previous results.

The key findings of this study demonstrated that osteogenesis‐related proteins, including BMP2, OPN, RUNX‐2 and Osterix, were regulated by miR‐93‐5p. BMP‐2 is an effective osteoinductive protein that can facilitate osteoblast differentiation and accomplish bone repair [31, 32]. Several studies have reported that BMP‐2 could serve as adjuvant therapy in ONFH surgical treatment [33, 34]. Another factor, OPN, is a key component in osteoclast attachment to bone during resorption. A recent study reported by Luukkonen *et al*. [35] observed that OPN was detected in areas of increased bone metabolism. We found that the expression level of BMP‐2 and OPN in the model + BMSCs/miR‐93‐5p inhibitor group was markedly higher than the model group, revealing miR‐93‐5p inhibition could enhance the ability of bone metabolism. Moreover, RUNX‐2 can regulate the formation and differentiation of BMSCs into osteoblasts [36]. In this study, inhibiting miR‐93‐5p could increase the expression of Runx‐2, suggesting that miR‐93‐5p might suppress the osteogenic differentiation of BMSCs. Osterix encodes a member of the Sp subfamily of Sp/XKLF transcription factors. It is considered a bone‐specific transcription factor, which is essential for osteoblast differentiation and bone formation [37]. Similarly, the overexpression of Osterix was observed in the miR‐93‐5p inhibitor group. Furthermore, a previous study has revealed that addition of exogenous BMP‐2 to osteogenic precursor cells (MC3T3‐E1) can promote the Runx‐2, OPN and osterix protein expression, as well as osteogenic formation, indicating that these genes jointly promote the differentiation of osteogenesis [38]. The combined results indicated that miR‐93‐5p regulated osteogenic differentiation by affecting the expression of BMP‐2, OPN, RUNX‐2 and Osterix.

To comprehensively explore the mechanism of miR‐93‐5p in BMSCs osteogenic differentiation, we investigated the association between BMP‐2/OPG/RANKL and miR‐93‐5p using *in vitro* experiments. We observed that BMP‐2 expression was significantly increased in the inducer + NC group and was dramatically suppressed in the inducer + miR‐93‐5p mimic group, which was consistent with the prior study [14]. The expression of OPG was significantly increased in the inducer + miR‐93‐5p NC and markedly decreased in the inducer + miR‐93‐5p mimic group. RANKL expression significantly declined in the inducer + miR‐93‐5p NC groups, but was increased by treatment with miR‐93‐5p mimic. A previous study has reported a close association between the OPG, as well as RANKL, and the development of ONFH [39]. Fu *et al*. found that proper mechanical stress could promote osteonecrosis recovery via the OPG/RANK/RANKL system [40]. These studies emphasized that OPG and RANKL might be potential therapeutic targets for ONFH. Taken together, we further revealed that miR‐93‐5p might regulate osteogenic differentiation via affecting the expression of BMP‐2, OPG and RANKL.

The concentrated implantation of BMSCs could relieve hip pain and prevent the progression of osteonecrosis [41]. Therefore, BMSCs are frequently used for treatment of ONFH, and the clinical effects have been recognized. In this study, the results indicated that compared with the BMSCs group, the miR‐95‐5p inhibitor group demonstrated a stronger effect on regulating osteogenic differentiation. We suggest miR‐93‐5p as a probable key regulator of osteogenic differentiation and as a potential therapeutic target for TIONF treatment.

## Conclusions

Overall, the results suggested that miR‐93‐5p had a significant biological effect on the osteogenic differentiation of BMSCs both *in* *vivo* and *in* *vitro*. Inhibition of miR‐93‐5p increased the expression of BMP‐2 mRNA and protein, indicating that miR‐93‐5p could function as osteogenic differentiation suppressors by reducing BMP‐2 expression in patients with TIONFH. In addition, we observed that miR‐93‐5p affected the OPN, RUNX‐2, Osterix, OPG and RANKL expression in the process of osteogenic differentiation. Our results provided evidence that miR‐93‐5p could be considered as a new therapeutic target for TIONFH. However, further studies on feasibility still need to be conducted before miR‐93‐5p can be targeted for clinical therapy.

## Conflict of interest

The authors declare no conflict of interest.

## Author contributions

YZ, WL and QW contributed to conception and design of the research. LZ, ZH and HW acquired the data. YZ and YL contributed to analysis and interpretation of data. ZZ and PL contributed to statistical analysis. YZ and WL obtained funding. PL and YF drafted the manuscript. YF, ZZ and QW contributed to revision of the manuscript for important intellectual content. All authors read and approved the final manuscript.

## Data Availability

All data generated or analyzed during this study are included in this published article.
